# Effects of Incubation Conditions on Cr(VI) Reduction by *c*-type Cytochromes in Intact *Shewanella oneidensis* MR-1 Cells

**DOI:** 10.3389/fmicb.2016.00746

**Published:** 2016-05-19

**Authors:** Rui Han, Fangbai Li, Tongxu Liu, Xiaomin Li, Yundang Wu, Ying Wang, Dandan Chen

**Affiliations:** ^1^School of Environment and Energy, South China University of TechnologyGuangzhou, China; ^2^Guangdong Key Laboratory of Agricultural Environment Pollution Integrated Control, Guangdong Institute of Eco-Environmental and Soil SciencesGuangzhou, China

**Keywords:** *c*-type cytochromes, *Shewanella oneidensis* MR-1, Cr(VI) reduction, intact cells, incubation conditions

## Abstract

It is widely recognized that the outer membrane *c*-type cytochromes (OM *c*-Cyts) of metal-reducing bacteria play a key role in microbial metal reduction processes. However, the *in situ* redox status of OM *c*-Cyts during microbial metal reduction processes remain poorly understood. In this study, diffuse-transmission UV/Vis spectroscopy is used to investigate the *in situ* spectral reaction of Cr(VI) reduction by *c*-Cyts in intact *Shewanella oneidensis* MR-1 cells under different incubation conditions. The reduced *c*-Cyts decreased transiently at the beginning and then recovered gradually over time. The Cr(VI) reduction rates decreased with increasing initial Cr(VI) concentrations, and Cr(III) was identified as a reduced product. The presence of Cr(III) substantially inhibited Cr(VI) reduction and the recovery of reduced *c*-Cyts, indicating that Cr(III) might inhibit cell growth. Cr(VI) reduction rates increased with increasing cell density. The highest Cr(VI) reduction rate and fastest recovery of *c*-Cyts were obtained at pH 7.0 and 30°C, with sodium lactate serving as an electron donor. The presence of O_2_ strongly inhibited Cr(VI) reduction, suggesting that O_2_ might compete with Cr(VI) as an electron acceptor in cells. This study provides a case of directly examining *in vivo* reaction properties of an outer-membrane enzyme during microbial metal reduction processes under non-invasive physiological conditions.

## Introduction

The biogeochemical cycles of many major and trace elements are driven by microbial redox processes. Dissimilatory metal-reducing bacteria (DMRB) are considered the most important microorganisms for controlling metal transformations under anoxic conditions. Typical strains of DMRB, such as the *Shewanella* and *Geobacter* species, have been widely investigated in terms of genetic diversity, structural and functional characterization of highly purified proteins, and traditional reductionist approaches (Beliaev et al., 2001, 2005; Kengo et al., 2010; Tremblay et al., 2012). *In vitro* reaction kinetics between metals and outer membrane *c*-type cytochromes (OM *c*-Cyts) of DMRB were studied using highly purified OM *c*-Cyts extracted from *Shewanella* species (Borloo et al., 2007; Ross et al., 2009; Belchik et al., 2011), and the roles of cytochromes (e.g., MtrC and OmcA) were characterized by examining the effects of mutant deletion. However, the purified proteins may behave differently from the protein complexes in live cells because the highly reactive enzymes may be easily changed during the purification (Nakamura et al., 2009). Hence, an *in vivo* study of the reaction between metals and *c*-Cyts in the live cells will allow a more comprehensive understanding of microbial metal reduction processes.

The electron transfer center of OM *c*-Cyts is a heme group, which has a large molar absorption coefficient. Therefore, spectroscopic methods have been applied to OM *c*-Cyts in living cells under physiological conditions (Nakamura et al., 2009; Liu et al., 2011). However, accurate measurement of heme groups in living cells is difficult due to the strong spectral interference from light scattering of cell surface. Fortunately, using a diffuse-transmission (DT) mode, this spectral interference was not observed in the absorption spectra of the multi-heme *c*-Cyts in whole cells (Nakamura et al., 2009). A number of recent studies used DT-UV/Vis spectroscopy to monitor heme groups in iron-/humic-reducing bacteria such as *Klebsiella pneumonia* L17 (Liu et al., 2014), *Shewanella putrefaciens* 200 (Zhang et al., 2014), *S. decolorationis* S12, *Aeromonas hydrophila* HS01 (Li et al., 2014), and *Leptospirillum ferrooxidans* (Blake and Griff, 2012). Recently, Wu et al. (2014) also employed DT-UV/Vis spectroscopy to analyze *in situ* spectral kinetics of electron shuttling reduction by *c*-Cyts in a living *S. putrefaciens* 200 suspension. *In situ* spectroscopy was also used to study the reaction between Fe(III) and *c*-Cyts in intact *L. ferrooxidans* under oxic conditions (Liu et al., 2014). In addition, Busalmen et al. (2008) reported an application of infrared spectroscopy in a whole-cell system in which the electron transfer between *Geobacter* and a gold electrode was monitored. It is true that there are some limitations about examining *c*-Cyts in intact cells by DT-UV/Vis spectroscopy. Using DT-UV/Vis spectroscopy, only the *c*-Cyts located on the very surface of cell outer-membrane can be measured directly by using this spectral method (Nakamura et al., 2009), but the *c*-Cyts located in periplasm and the cytoplasmic membrane were unlikely to be measured as the light was not able to penetrate the membrane. Hence, the *c*-Cyts measured in the cell suspension can only represent the outer membrane (OM) *c*-Cyts but not the total *c*-Cyts. In addition, the OM *c*-Cyts include a series of different proteins, such as MtrC and OmcA in MR-1, but it is difficult to differentiate the roles of MtrC and OmcA from those of other *c*-Cyts by just using the DT UV-Vis spectral method. Despite the aforementioned limitations of DT-UV/Vis spectroscopy, this method is promising to study the *in situ* behavior of OM *c*-Cyts in intact DMRB cells.

Chromium is toxic and is designated a priority pollutant in many countries (Middleton et al., 2003). Microbial reduction of Cr(VI) to Cr(III) can be considered as a way to remediate Cr(VI) contaminations because Cr(III) is less water-soluble, less mobile, and much less toxic than Cr(VI) (James, 1996; Barnhart, 1997). A variety of DMRB have been reported to reduce Cr(VI) to Cr(III) (Puzon et al., 2005; Cheung and Gu, 2007; He et al., 2011). Cr(VI)-reducing bacteria use electron transport systems containing cytochromes in their OM to reduce Cr(VI) derivatives during anaerobic respiration (Myers et al., 2000; Ahemad, 2014). *S. oneidensis* MR-1 is considered a model organism for metal reduction as it is able to reduce a variety of metals. Recent studies have shown that MR-1 can reduce Cr(VI) to Cr(III) as a terminal electron acceptor under anoxic conditions with OM *c*-Cyts (e.g., OmcA and MtrC) serving as terminal reductases of Cr(VI) (Myers and Myers, 2001; Belchik et al., 2011; Wang et al., 2013). However, the molecular scale reaction mechanism of the Cr(VI) and *c*-Cyts remains poorly characterized under non-invasive physiological conditions. Direct photometric studies of intact cells and Cr(VI) will allow elucidation of these *in vivo* reaction mechanisms.

The microbial Cr(VI) reduction can be influenced by different environmental factors, such as Cr(VI) concentration, temperature, bacterial cell density, electron donor, pH, oxygen, and the presence of other metal ions (Wang and Xiao, 1995; Dey et al., 2014). Past studies (Wang and Xiao, 1995; Zakaria et al., 2007; Dey et al., 2014) mainly focused on effects of incubation factors on the apparent Cr(VI) reduction and rarely investigated the effects of incubation factors on the redox status of *c*-Cyts in the OM of DMRB, which play a key role in microbial Cr(VI) reduction. *In situ* spectral kinetics of Cr(VI) reduction by *c*-Cyts in live cells under different environmental conditions should provide a more fundamental understanding of the molecular-level mechanisms.

Actually, the previous studies on DT-UV/Vis spectroscopy just focused on qualifying the *c*-Cyts in the living cells (suspension or biofilm) but did not conduct the quantification of *c*-Cyts in the living cells. In this study, the Cr(VI) reduction was investigated in a living cell suspension of *S. oneidensis* MR-1 on DT-UV/Vis spectroscopy with the objectives: (1) to quantify the *c*-Cyts in the living MR-1 cell suspensions by DT-UV/Vis spectroscopy, (2) to directly examine *in situ* spectral kinetics of Cr(VI) and *c*-Cyts in intact cells of MR-1, and (3) to evaluate the effects of different incubation conditions, i.e., cell density, initial Cr(VI) concentration, Cr(III) concentration, pH, temperature, electron donors and oxygen on the *in vivo* reaction between Cr(VI) and *c*-Cyts.

## Materials and Methods

### Materials

*Shewanella oneidensis* MR-1 was isolated from anoxic sediments of Lake Oneida, NY (Myers and Nealson, 1988) and purchased from MCCC (Marine Culture Collection of China, China). The strain was grown aerobically overnight as batch cultures in Luria-Bertani (LB) medium (10 g L^-1^ NaCl, 5 g L^-1^ yeast extract, 10 g L^-1^ tryptone) to exponential phase at 30°C with shaking at 180 rpm. The cells were subsequently washed and diluted before the *in situ* spectral kinetic experiments. All chemicals used in the experiments were reagent grade or better. Water for all experiments was supplied from a Milli-Q reference ultraviolet (UV)-water system. Horse heart cytochrome *c* was obtained from Sigma–Aldrich (China). Cr(VI) stock solution was prepared by dissolving potassium dichromate (K_2_Cr_2_O_7_; Sigma–Aldrich) in UV-water. Chromium chloride hexahydrate (CrCl_3_⋅6H_2_O; 98.45%, AR, Aladdin, China) was used as the source of Cr(III). A 30 mM solution of 4-(2-hydroxyethyl)piperazine-1-ethanesulfonic acid (HEPES, Sigma–Aldrich) adjusted to pH 7 with sodium hydroxide was used as the buffer in all experiments. Stock solutions of sodium lactate (1.0 M) were also prepared in UV-water for use in the experiments. All stock solutions were stored at 4°C before use.

### Quantification of *c*-Cyts in a Live-Cell Suspension

The strain was aerobically inoculated in Luria-Bertani (LB) for 16 h in a shaker at 180 rpm and 30°C and harvested by centrifugation at 8,000 × *g* for 10 min at 4°C for three times after being washed and re-suspended using HEPES buffer when it approached the exponential phase. The cell suspension in HEPES buffer was purged with 100% N_2_ for 30 min, and then the suspension with lactate (20 mM) as electron donor was added to a rectangular quartz cuvette with an optical path length of 1.0 cm for measurement before sealing in Anaerobic Chamber. The horse heart cytochrome *c* was used as a standard (Picardal et al., 1993) to quantify the *c*-Cyts in livin*g* MR-1 cell suspension. Different concentrations of horse heart cytochrome *c* were measured by a UV/Vis spectrophotometer (TU-1901 Beijing, China) equipped with an IS19-1 integrating sphere reflectance attachment, using a 10-mm optical path of dish, with 1 nm scan interval, and 1.0 nm s^-1^ sweep speed from 300 to 600 nm. A difference of millimolar extinction coefficients (Δ𝜀) of 21.4 mM^-1^ cm^-1^ (Supplementary Figure S1) for reduced and oxidized forms of meso-IX pyridine hemochrome was used for quantification of heme *c* (Picardal et al., 1993). Using the spectral method, only the *c*-Cyts located on the very surface of cell outer-membrane can be measured directly, but the *c*-Cyts which located in periplasm and the cytoplasmic membranes were unlikely to be measured as the light was not able to penetrate the membrane. Hence, the *c*-Cyts measured in the cell suspension can only represent the outer membrane *c*-Cyts. In addition, we were not able to differentiate the specific roles of individual proteins from those of other *c*-Cyts by just using the DT UV-Vis spectral method, but we just examined the outer-membrane heme-containing molecules as the bulk OM *c*-Cyts in intact DMRB cells.

### Procedures for Cr(VI) Reduction by MR-1

MR-1 was grown aerobically in LB at 30°C for 16 h. The suspension was subsequently centrifuged at 7000 × *g* for 10 min at 4°C, and the pellets were washed with sterile HEPES buffer (30 mM, pH 7.0) three times. Cells were then transferred to an Anaerobic Chamber (DG250, Don Whitley Scientific, England) with H_2_: N_2_ = 4:96 and resuspended in the same buffer for a final concentration of 1.07 × 10^12^ cells mL^-1^. This cell suspension was added to a rectangular quartz cuvette with an optical path length of 1.0 cm. Sodium lactate (20 mM) was added to the suspension as an electron donor. Cr(VI) was added as the sole electron acceptor at concentrations ranging from 20 to 1000 μM. The cuvette was subsequently sealed and taken out of the Anaerobic Chamber. Two control solutions that lacked cells or contained autoclaved cells were used for each Cr(VI)-reduction assay to examine the effects of any abiotic factors or the biosorption of Cr(VI) by the cell mass on Cr(VI) reduction, respectively. The cell suspension before Cr(VI) addition was also measured. Once Cr(VI) was added, spectra were then taken using the DT-UV/Vis spectrophotometer by scanning at different intervals. The specific peaks appear at 372 nm for Cr(VI) and at 552 nm for reduced cytochrome (*c*-Cyt_red_). A pseudo first-order model can be applied to describe the kinetics of Cr(VI) reduction by MR-1. There was no replicate in the spectral kinetic experiments for *c*-Cyts and Cr(VI). The error bars of *k*-values represent the standard error of different *k*-values calculated from linear fitting by Origin software.

### Cr(VI) Reduction by MR-1 under Different Incubation Conditions

The effect of additional Cr(III) on Cr(VI) reduction by MR-1 cells was investigated using different initial concentrations of Cr(III) (50–1000 μM). To investigate the effect of MR-1 initial cell density on chromate reduction, a series of densities of MR-1 cells ranging from 2.68 × 10^11^ cells mL^-1^ to 2.14 × 10^12^ cells mL^-1^ were added to the system. Effects of different pH values (6.0–8.0) and temperatures (20–40°C) on the Cr(VI)-reducing capability of viable MR-1 cells were determined. The effect of pH was determined using sterile PIPES (piperazine-N, N-bis-2-ethanesulphonic) buffer (30 mM) and HEPES buffer (30 mM). In both cases, an initial concentration of 160 μM of Cr(VI) was used as the sole electron acceptor. Cr(VI) reduction by viable cells of MR-1 was studied in the presence of various electron donors, including 20 mM of D-lactose, formate, lactate and sucrose from the stock solutions (1 M). The effect of oxygen concentration on Cr(VI) reduction by MR-1 cells was investigated with different concentrations of dissolved oxygen (DO), including 1.0, 3.0, 4.0, 16, 26, 33, and 68 μM.

### Cr(VI) Reduction by *c*-Cyts in Various Intact Mutant Cells

To examine the electron transport chain of MR-1, the Cr(VI) reduction was examined by using MR-1 with *c*-Cyt deletion mutants including Δ*mtrC*, Δ*mtrF*, Δ*mtrA*, Δ*mtrD*, Δ*omcA*, and Δ*cymA* (Hartshorne et al., 2009; Coursolle and Gralnick, 2010; Shi et al., 2012). The mutant strains used in this study were provided by Professor Haichun Gao in Zhejiang University, and the relevant information was provided in the reference (Gao et al., 2010). MR-1 wild type (wt) and various mutants were grown aerobically in LB at 30°C for 16 h. The suspension was subsequently centrifuged at 7000 × *g* for 10 min at 4°C, the pellets were washed with sterile HEPES buffer (30 mM, pH 7.0) three times and subsequently used for *in situ* spectral kinetic experiments.

### Characterization of MR-1 Cells and Cr Species

After approximately 60 min of incubation, the cells were centrifuged at 7000 × *g* for 4 min and flash-frozen with liquid nitrogen before RNA was extracted using TRIzol reagent (Invitrogen, USA). Reverse transcription PCR and real-time quantitative PCR were then used to measure the amount of 16S rRNA gene. The obtained bacterial RNA was reverse transcribed using a PrimeScript^TM^ II 1st strand cDNA Synthesis Kit (Takara, Shiga, Japan) according to the instructions of the manufacturer. Quantification of transcripts of bacterial 16S rRNA was determined by the iQ^TM^5 Real-Time PCR Detection System (Bio-Rad, USA) and using the SYBR Green I detection method. The qPCR System using the Eub338 (ACTCCTACG GGAGGCAGCAG; Lane, 1991) and Eub518 (ATTACCGCGGCTGCTGG; Muyzer et al., 1993) primer pair. Each 20 μL reaction solution contained the following: 1 μL of template cDNA (1–10 ng), 10 μL of 2× IQ SYBR^TM^ Green Supermix (Bio-Rad, USA), 0.2 μM of each primers. PCR conditions were 5 min at 95°C, followed by 40 cycles of 94°C for 20 s, 55°C for 20 s, and 72°C for 30 s (Muyzer et al., 1993). Per DNA sample and the appropriate set of standards were run in triplicates. The qPCR calibration curves were generated with serial triplicate 10-fold dilutions of plasmid DNA. The plasmid pGEM-T Easy Vector (Promega, Madison, WI, USA) contained the cloned target sequences. Plasmid DNA was extracted using an EZNA Plasmid Mini Kit I (Omega Bio-Tek, Doraville, GA, USA) and the concentration was quantified by the Qubit 2.0 Fluorometer (Invitrogen, NY, USA). Target copy numbers for each reaction were calculated from the standard curves (Pfaﬄ, 2001). The experiments for 16s rRNA were conducted in triplicate, and the mean values and error bars were derived from average and standard deviation values calculated from three repeats.

To identify the location and valences of the reduced product, MR-1 cells associated with the reduced product after incubation were characterized by scanning electron microscopy-energy dispersive X-ray (SEM-EDX) and X-ray photoelectron spectroscopy (XPS). For SEM-EDX analysis, the bacterial cells associated with reduced product were washed with phosphate buffer (10 mM, pH = 7.5) and fixed in 2.5% (v/v) glutaraldehyde. The sample was then dried with ethanol under ambient conditions, mounted on an aluminum stub and then coated with gold. Images and energy-dispersive X-ray spectroscopy (EDX) were taken using an FEI Quanta 400F thermal-field emission environmental SEM (FEI, Hillsboro, OR, USA) with an Oxford INCA EDX. For the XPS analysis, the cells were exposed to 1000 μM Cr(VI) for 10 h, while the cells under the same conditions without Cr(VI) served as a control. The cells were then separated by centrifugation at 10,000 × *g* at 4°C for 5 min. The pellets were washed with deionized water and freeze-dried overnight at -42°C. The sample was examined by an ESCALAB 250 XPS (Thermo-VG Scientific, USA).

## Results

### Spectral Monitoring of Cr(VI) Reduction by *c*-Cyts in Intact Cells

**FIGURE 1:**
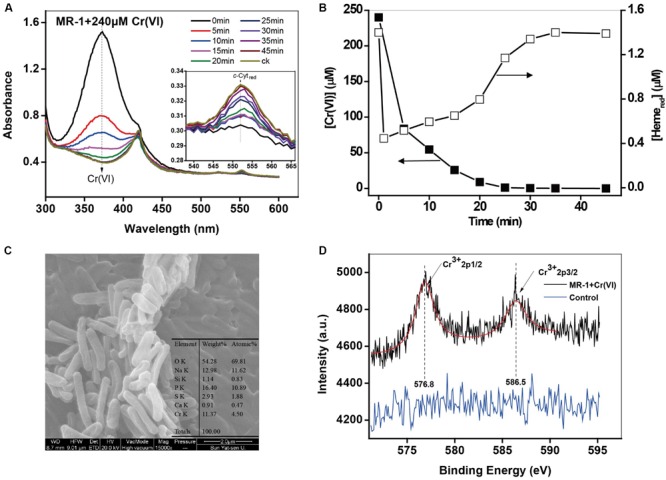
**(A,B)** The *in situ* kinetic spectra of the intact MR-1 cell suspensions with 240 μM Cr(VI) under anoxic conditions. The initial cell density of MR-1 was 1.07 × 10^12^ cells mL^-1^. The peak at 372 nm corresponds to Cr(VI) and the peak at 552 nm corresponds to Heme_red_. Characterization of the reduced products: **(C)** SEM-EDS analysis of the products of Cr(VI) reduction by MR-1. **(D)** XPS analysis of the products in the control of killed MR-1 cells.

The spectra in **Figure 1A** clearly show changes of specific peaks at 372 nm, corresponding to Cr(VI), and at 552 nm, corresponding to Heme_red_. The heights of these peaks were extracted and plotted in **Figure 1B**. While Cr(VI) decreased substantially, the Heme_red_ transiently dropped to a low level at the very beginning (stage 1: consumption) and then gradually increased to the initial level (stage 2: recovery). It has been widely reported (Ramirez-Diaz et al., 2008; Gnanamani and Kavitha, 2010; Belchik et al., 2011) that the transformation from Cr(VI) to Cr(III) is the dominant pathway of microbial Cr(VI) reduction (Faisal and Hasnain, 2004). The redox potentials of Cr(VI)/Cr(III) and *c*-Cyt_ox_/*c*-Cyt_red_ at pH 7 were 1.2 and 0.25 V, respectively (Morel and Hering, 1993), indicating that the reaction between *c*-Cyts and Cr(VI) was thermodynamically feasible. To further prove the direct redox reaction between Cr(VI) and *c*-Cyts, the proteins have been extracted, and similar patterns of oxidized (*c*-Cyt_ox_) and reduced *c*-Cyts (*c*-Cyt_red_) were observed in the spectra of whole cells and extracted OM proteins of MR-1 (Supplementary Figure S2A). After Cr(VI) was added to reduced OM proteins, the peak intensity at 552 nm rapidly decreased, indicating that the reaction between *c*-Cyt_red_ and Cr(VI) definitely occurred. Therefore, this result can provide a direct evidence for the electron transfer from *c*-Cyt_red_ to Cr(VI). The SEM images showed that obvious white precipitation was generated on the surfaces of the Cr(VI)-treated cells (**Figure 1C**). While no morphological change of the cells was observed, presumably due to the short period of experiments, a chromium signal was found in the precipitation in EDX analysis with a Cr element composition of 11.37%. The XPS results (**Figure 1D**) show two distinct peaks: 586.5 eV (Cr 2p1/2) and 576.8 eV (Cr 2p3/2), representing Cr(III) in the form of a Cr_2_O_3_-like species (Biesinger et al., 2004; Manning et al., 2007; Dong et al., 2013). A control with Cr(III) and MR-1 under anoxic conditions was also conducted (Supplementary Figure S2B), with results indicating that the Cr(III) reduction products did not influence the heme measurements. Moreover, the cells under oxic conditions showed that oxidized heme (Heme_ox_) had no reactivity for Cr(VI) removal (Supplementary Figure S2C). Hence, it is reliable to directly measure Cr(VI) and Heme_red_ using the DT spectra.

### Cr(VI) Reduction by *c*-Cyts in Intact Mutant Cells

**FIGURE 2:**
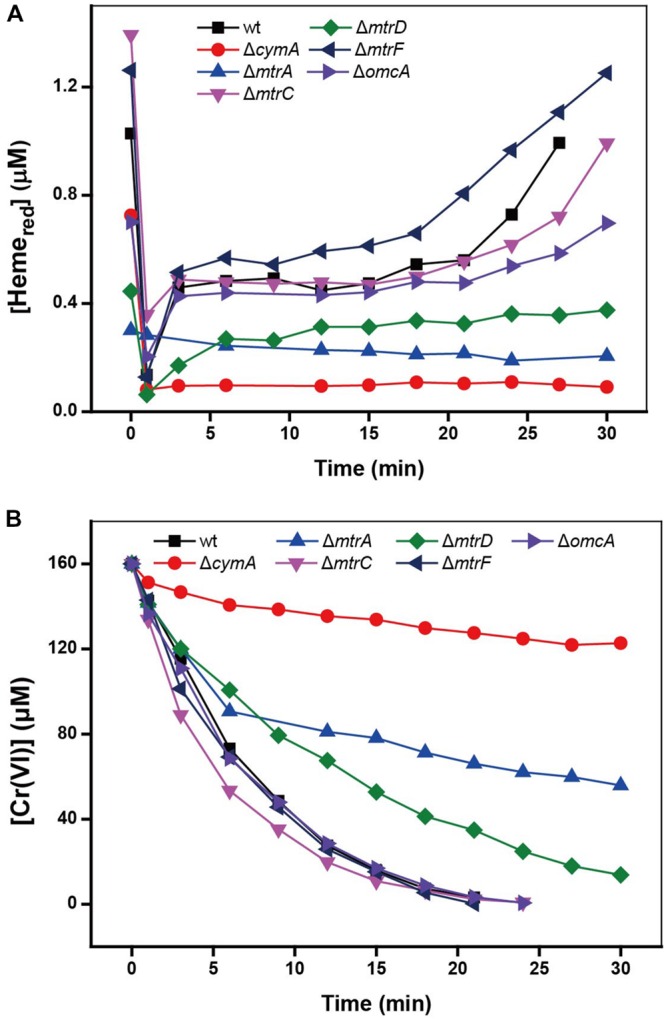
**The kinetics of Cr(VI) reduction by MR-1 wild type and the mutants without MtrA, MtrC, MtrD, MtrF, OmcA, and CymA. (A)** Cr(VI) reduction; **(B)** Heme_red_ reduction. All experiments conducted with Cr(VI) 160 μM, MR-1:1.07 × 10^12^ cells mL^-1^, sodium lactate is 20 mM.

Results in **Figures 2A,B** with various mutants showed that, compared to the results for the wild type (wt), the mutants Δ*cymA* and Δ*mtrA* exhibited distinctly low reduction rates of Cr(VI) and Heme, demonstrating their specific role in controlling the electron transport chain for Cr(VI) reduction. It was reported that CymA can oxidize the quinol in the inner membrane and then transfer the electrons to MtrA through other periplasmic proteins (Shi et al., 2012). Deletion of CymA evidently hindered the MR-1 cell to use the terminal electron acceptors (Schwalb et al., 2003). A mutant lacking MtrA also exhibited attenuated reduction of external electron acceptors, demonstrating its critical role in metal reducing processes (Beliaev et al., 2001). The MtrC and OmcA are localized on bacterial cell surfaces (Belchik et al., 2011), but the deletion of MtrC or OmcA had rarely effect on reduction rates of Cr(VI) and Heme, suggesting the existence of multiple Cr(VI) reduction pathways, which is consistent with the existence in the MR-1 genome of numerous MtrABC paralogs (Coursolle and Gralnick, 2010).

### Effect of Initial Cell Densities

**FIGURE 3:**
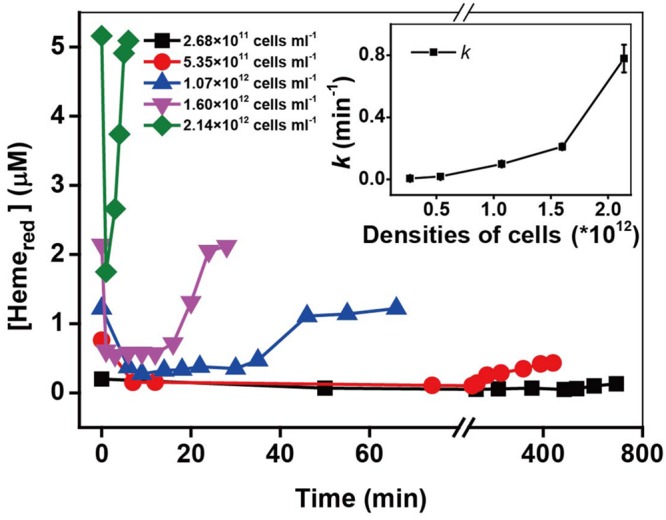
**The kinetics of Cr(VI) reduction by *c*-Cyts in intact MR-1 cells with densities ranging from 2.68 × 10^11^ cells mL^-1^ to 2.14 × 10^12^ cells mL^-1^.** The relationship between the pseudo-first-order rate constants *k* and the initial MR-1 cell concentration (insert chart). All experiments were conducted with 160 μM Cr(VI) and 20 mM sodium lactate.

The heme concentration is directly dependent on the cell densities of MR-1 cell suspension, therefore, the cell densities might have a great influence on Cr(VI) reduction by MR-1. Kinetic results in Supplementary Figure S3 and **Figure 3** show that the reduction rate of Cr(VI) increased proportionally with an increase in cell densities. Similar behaviors have been reported with *Arthrobacter* sp. SUK 1201 (Dey et al., 2014), *Pseudomonas* CRB5 (McLean et al., 2000), and *Bacillus sphaericus* AND 303 (Pal and Paul, 2004). Simultaneously, the higher cell density induced faster Heme_red_ recovery. Here we provide a direct evidence of Heme_red_ recovery by measuring *in situ* kinetics of *c*-Cyts in intact MR-1 cells.

### Effect of Initial Cr(VI) Concentrations

**FIGURE 4:**
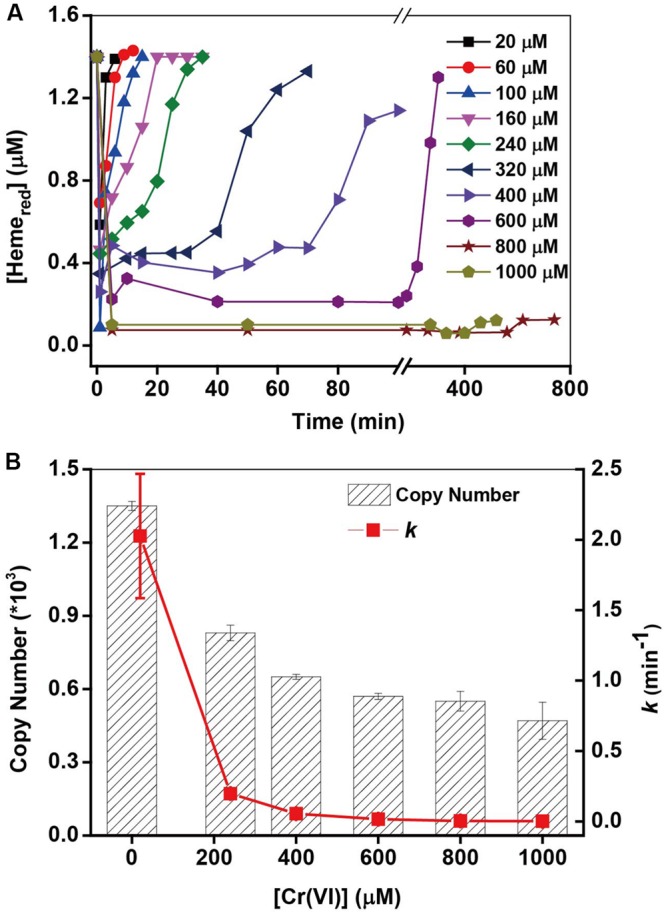
**(A)** The kinetic relationship between the pseudo-first-order rate constants *k* and the initial Cr(VI) concentration on Cr(VI) reduction by MR-1 (line chart). **(B)** The 16S rRNA copy numbers from MR-1 incubated with different concentrations of Cr(VI) for 60 min (column), (mean ± SD, *n* = 3). [Cr(VI)] ranged from 20 to 1000 μM, MR-1: 1.07 × 10^12^ cells mL^-1^.

The kinetics of Cr(VI) and *c*-Cyts are examined (Supplementary Figure S4) under different initial Cr(VI) concentrations. The pseudo-first-order rate constant (*k*) of Cr(VI) reduction in **Figure 4A** decreased greatly with an increase in initial Cr(VI) concentrations. For example, the *k*-value of 20 μM Cr(VI) (2.027 min^-1^) is nearly 700-fold higher than that of 1000 μM Cr(VI) (0.003 min^-1^). Simultaneously, the Heme_red_ changed accompanying the changes of Cr(VI) (**Figure 4B**), showing that while the consumption of Heme_red_ in stage (i) of all the treatments was nearly the same, the recovery of Heme_red_ in stage (ii) became increasingly slow with an increase in initial [Cr(VI)]. The slower recovery of Heme_red_ might account for the low *k*-values of Cr(VI) reduction with high initial [Cr(VI)]. It has been reported that Cr(VI) is toxic to metal-reducing bacteria and has resulted in a loss of metal-reduction activity (Cervantes et al., 2001). Therefore, to examine the toxicity of Cr(VI) to MR-1 cells, 16S rRNA analysis of MR-1 was carried out after MR-1 was incubated with different Cr(VI) concentrations for 60 min. The results in **Figure 4B** show that the 16S rRNA copy numbers decreased greatly with increasing Cr(VI) concentrations, suggesting that the Cr(VI)-reducing capacity of MR-1 declined probably due to the toxicity at higher [Cr(VI)], which is consistent with other well-documented results (Sugden et al., 2001; Middleton et al., 2003; Viamajala et al., 2003). This studies suggested that while the high concentrations of Cr(VI) and its reduced product Cr(III) may cause toxicity to the intact cells, this might be an effective and environmental-friendly method for the bioconversion of Cr(VI) with low concentrations.

### Effect of Added Cr(III) Concentrations

**FIGURE 5:**
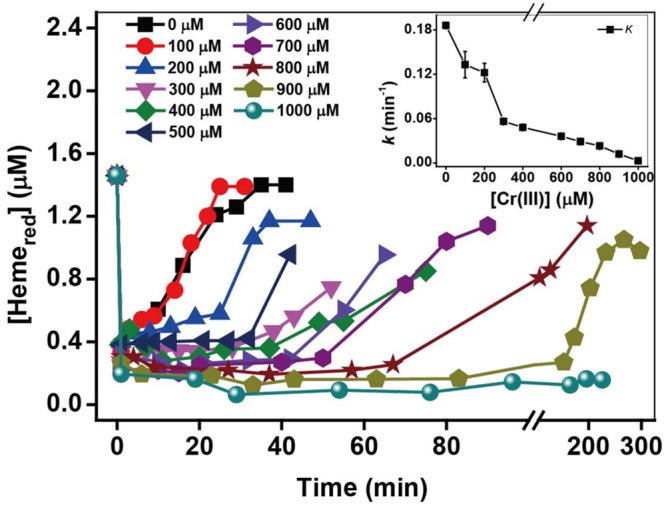
**The relationship between the pseudo-first-order reaction rate constants *k* and Cr(III) concentration.** [Cr(III)] ranged from 100 to 1000 μM (insert chart). All experiments were conducted with 160 μM Cr(VI), MR-1: 1.07 × 10^12^ cells mL^-1^, and 20 mM sodium lactate.

Kinetic results in the presence of 50–1000 μM Cr(III) in Supplementary Figure S5 and *k*-values in **Figure 5** show that the Cr(VI) rates decreased substantially with an increase in Cr(III) concentration, indicating that the inhibitory effects of Cr(III) increased gradually with increasing [Cr(III)]. The kinetics of Heme_red_ in **Figure 5** show that the recovery of Heme_red_ in the presence of 100 μM Cr(III) is almost the same as that of the control in the presence of Cr(III). However, the extent of Heme_red_ recovery decreased with increasing Cr(III) concentrations from 200 to 1000 μM, indicating that Cr(III) might be toxic to microorganisms due to its inhibition of the transformation of Heme_ox_ to Heme_red_. The above results suggest that the inhibition by added exogenous Cr(III) might result from the combination of Cr(III) and Heme_ox_ in OM of MR-1, which may prevent the reaction of Heme_ox_ to Heme_red_, thus slowing the rate of Cr(VI) reduction. Also, the added Cr(III) as freshly dissolved CrCl_3_ appeared toxicity, which be associated with extracellular interactions and caused an ultimately lethal cell morphology (Parker et al., 2011). The addition of CrCl_3_ to *Shewanella* species causes an abrupt drop in cell survival (Bencheikh-Latmani et al., 2007). Hence, the results here about the effect of Cr(III) concentrations might be not only related to the combination of Cr(III) and Heme_ox_ in MR-1 OM but also related to the toxic effect of freshly dissolved CrCl_3_.

The aforementioned results are mainly based on the effects of endogenous factors, added reactants [Cr(VI) and *c*-Cyts] and products [Cr(III)], and *c*-Cyts recovery. These factors can also be influenced by external incubation factors, such as suspension pH, temperature, types of electron donors, and oxygen concentrations. The *in situ* spectral kinetics of Cr(VI) reduction and *c*-Cyts recovery were further investigated in terms of these external factors.

### Effect of pH

**FIGURE 6:**
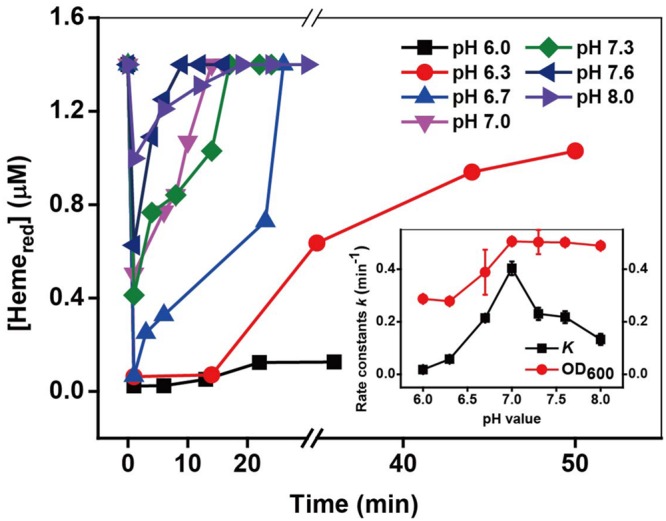
**The kinetics of Cr(VI) reduction by *c*-Cyts in intact MR-1 cells at pH conditions ranging from pH 6.0 to pH 8.0.** The relationship between the pseudo-first-order reaction rate constants *k* and pH conditions ranging from pH 6.0 to pH 8.0 (insert chart). All experiments were conducted with 160 μM Cr(VI), MR-1: 1.07 × 10^12^ cells mL^-1^, and 20 mM sodium lactate.

Cr(VI) reduction rates are strongly dependent on pH (Wang et al., 1989; Philip et al., 1998; Fein et al., 2010). The acidity may affect the Cr(VI) reduction through effects on the properties of the MR-1 and Cr(VI) solution, such as the growth and metabolism of cells and the speciation of Cr(VI). The kinetic results under different pH in Supplementary Figure S6 and **Figure 6** show that the optimal Cr(VI) reduction rate was obtained at pH 7; however, the achieved recovery of Heme_red_ increased with an increase in pH from 6 to 8. As the pH may influence the growth of cells, the cell density the OD_600_ value was measured at the end of each reaction. The results show that the maximum OD_600_ value was also obtained at pH 7.0. This behavior is consistent with the fastest pH dependent-Cr(VI) reduction via metabolic enzymatic reactions occurring at pH 7.0 (Wang et al., 1989; Shen and Wang, 1994a; Philip et al., 1998).

### Effect of Temperature

**FIGURE 7:**
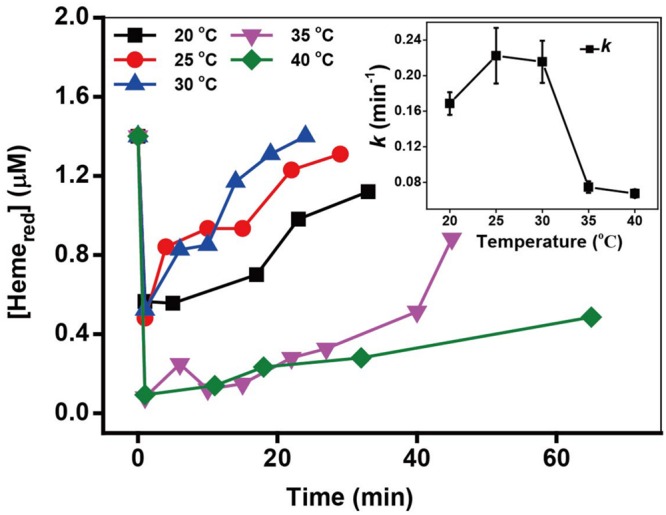
**The kinetics of Cr(VI) reduction by *c*-Cyts in intact MR-1 cells at temperatures ranging from 20 to 40°C.** The relationship between the pseudo-first-order rate constants *k* and temperatures ranging from 20 to 40°C (insert chart). All experiments were conducted with 160 μM Cr(VI), MR-1: 1.07 × 10^12^ cells mL^-1^, and 20 mM sodium lactate.

The incubating temperature is also an important factor affecting the cell growth and thus influencing the microbial Cr(VI) reduction. The kinetics of Cr(VI) reduction and the transformation of Heme_red_ in (Supplementary Figure S7 and **Figure 7**) revealed that the optimal Cr(VI) reduction rates and Heme_red_ recovery were obtained at 25–30°C. It has previously been reported that that biological activity of MR-1 is optimal at 30°C (Burgos et al., 2008).

### Effect of Different Electron Donors

**FIGURE 8:**
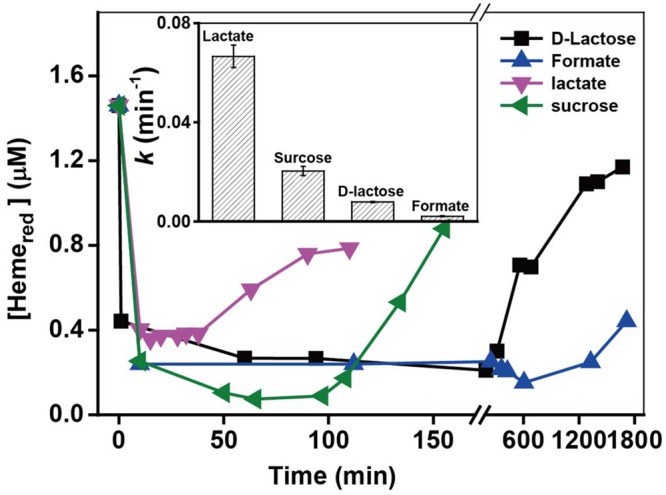
**The relationship between the pseudo-first-order rate constants *k* and added electron donors (insert chart).** All experiments were conducted with 160 μM Cr(VI), MR-1: 1.07 × 10^12^ cells mL^-1^, and 20 mM of the electron donor specified.

MR-1 can utilize a wide range of various carbon sources as electron donors, while the utilization efficiency of those electron donors are quite different, which might further influence the metabolic processes. The heme transformation rate may be associated with the intracellular electron transfer occurring via the cell metabolism, as electron donors may control metabolic processes and ultimately affect the intracellular electron transfer to Heme_ox_. The kinetic results with different electron donors in Supplementary Figure S8 and *k*-values in **Figure 8** show that the added electron donors affected the reduction rates of Cr(VI) in the following order: lactate > sucrose > D-lactose > formate. The *k*-value with lactate (0.0666 min^-1^) was almost 37 times higher than that in the presence of formate (0.0021 min^-1^). **Figure 8** shows that the ranking order of Heme_red_ recovery is consistent with that of Cr(VI) reduction. The fastest recovery of Heme_red_ occurred in the presence of lactate, while a barely detectable amount of Heme_ox_ was reduced in the presence of formate. The effects of electron donors on the reduction of Cr(VI) and Heme_red_ might be due to different utilization rates of electron donors by MR-1; this phenomenon was also reported in previous studies of Fe(III) reduction by MR-1 (Petrovskis et al., 1994; Park and Kim, 2001). Lactate was the most efficient carbon source for Cr(VI) reduction by MR-1. Thus, lactate is the most favorable of the tested carbon sources for facilitating the transformation from Heme_ox_ to Heme_red_ and the most efficient rate of Cr(VI) reduction.

### Effect of Oxygen

**FIGURE 9:**
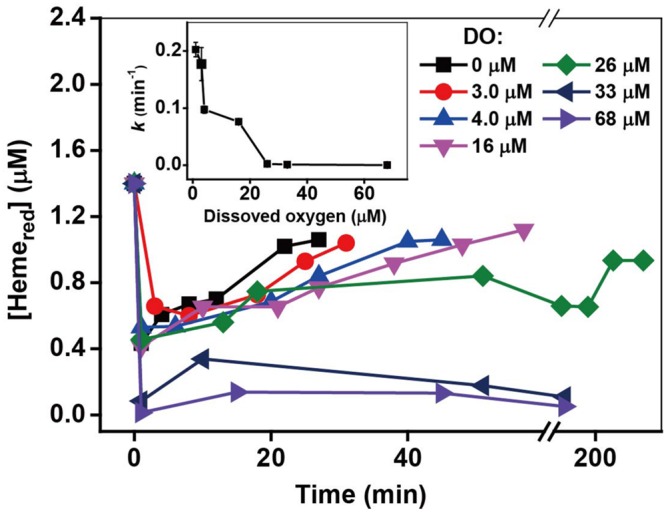
**The relationship between the pseudo-first-order rate constants *k* and O_2_ concentration.** The kinetics of Cr(VI) reduction by *c*-Cyts were measured in intact MR-1 cells at dissolved oxygen concentrations ranging from 0 to 68 μM (insert chart). All experiments were conducted with 160 μM Cr(VI), MR-1: 1.07 × 10^12^ cells mL^-1^, and 20 mM sodium lactate.

The *Shewanella* strain is a facultative anaerobic bacterium; thus, oxygen not only influences the metabolic rates but also acts as an electron acceptor that competes with Cr(VI) during the Cr(VI) reduction by MR-1 (Ohtake et al., 1990; Viamajala et al., 2004). It was reported that there was immediate cessation of growth upon addition of Cr(VI) in early- and mid-log-phase cultures under anaerobic conditions, while addition of Cr(VI) to aerobically growing cultures resulted in a gradual decrease of cell growth rate (Viamajala et al., 2004). Kinetic results with different oxygen concentrations are shown in Supplementary Figure S9 and *k*-values are shown in the insert of **Figure 9**. The control, which lacked oxygen, had the highest efficiency of Cr(VI) reduction, while an obvious inhibitory effect was observed in the presence of oxygen and the inhibitory effect increased substantially with increasing oxygen. Indeed, the Cr(VI) reduction capacity even disappeared with [O_2_] ≥ 26 μM. Consistent with Cr(VI) reduction, the reduction of Heme_ox_ to Heme_red_ was stable for [O_2_] from 0 to 16 μM, and then decreased with the increase of [O_2_] from 16 to 68 μM. The above results show that although the added oxygen might enhance the metabolic processes and increase cell growth, oxygen likely plays a key role in the inhibitory effects on Cr(VI) reduction.

## Discussion

In the process of Cr(VI) reduction by *c*-Cyts of intact MR-1 cells, the electron transfer pathway can be concisely divided into two steps: (i) intracellular electron transport from electron donor to OM *c*-Cyts and (ii) electron transfer from *c*-Cyts to Cr(VI). The step-by-step potential losses (Wu et al., 2014) can drive electron flow from electron donor via *c*-Cyts to Cr(VI), resulting in Cr(VI) reduction. Therefore, the current study only focuses on the Cr(VI) reduction by *c*-Cyts. In Step (i), the electron donor (lactate) can be utilized by MR-1 with concomitant intracellular electron transport from lactate to OM *c*-Cyts, resulting in the redox transformation of Heme_ox_ to Heme_red_ as shown in Eq. 1.

(1) $$C_{3}H_{5}O_{3}^{-}+2H_{2}{O + 4Heme}_{\mathrm{ox}}\to {4Heme}_{\mathrm{red}}+C_{2}H_{3}O_{2}^{-}+{\mathrm{HCO}}_{3}^{-}+5H^{+}$$

The Heme_ox_ and Heme_red_ represent the Fe(III)-associated and Fe(II)-associated hemes, respectively, and hence the redox transformation from Heme_ox_ to Heme_red_ can be considered as an one-electron transfer reaction. In Step (ii), Heme_red_ directly transfers electrons to Cr(VI) by generating Cr(III) when Cr(VI) is in contact with OM of MR-1.

(2) $${3Heme}_{\mathrm{red}}+\mathrm{Cr}(\mathrm{VI})\to \;{3Heme}_{\mathrm{ox}}+\mathrm{Cr}(\mathrm{III})$$

It was reported that the reaction rate constants (Eq. 2) are 3.5 × 10^4^ M^-1^ s^-1^ for MtrC and 2.5 × 10^5^ M^-1^ s^-1^ for OmcA (Belchik et al., 2011), so the transient oxidation of the outer membrane cytochromes occurred due to the fast molecular reaction between Heme_red_ and Cr(VI), which was also supported by the SEM results with obvious Cr(III) white precipitation on the surfaces of the Cr(VI)-treated cells.

In addition, from the results of Cr(VI) reduction by MR-1 mutants, the electron transfer pathway of reaction between Cr(VI) and c-Cyts of MR-1 is likely to be the typical Mtr respiratory pathway which is required for the reduction of metals and electrodes (Coursolle and Gralnick, 2010), suggesting that the outer-membrane enzyme-induced extracellular electron transfer might dominate the Cr(VI) reduction by MR-1. While the roles of those mutants in **Figure 2A** did not provide new interpretation in light of previous knowledge, but we go further with using DT UV-Vis spectral approach to measure the real-time changes of the redox status of *c*-Cyts in **Figure 2B**. From this aspect, the information about the *c*-Cyts in intact cells can be a new complement to the previous interpretation about the roles of various mutants.

It was reported that many microorganisms were capable of secreting high molecular mass polymers that can either be released into the surrounding environment (extracellular polysaccharides, exopolysaccharides) or remain attached to the cell surface (capsular polysaccharides; Ozturk and Aslim, 2008), and some quinone-compounds or flavin-compounds can play a role of shuttling electrons from cells to terminal electron acceptors (Marsili et al., 2008). Hence, besides the direct electron transfer from c-Cyts to Cr(VI), the electron shuttle-mediated Cr(VI) reduction was also possible in Cr(VI) reduction by MR-1. Generally, these two types of polysaccharides can be separated by centrifugation, with those remaining in the supernatant being soluble EPS (Sheng et al., 2010). The strain used in this study was harvested by centrifugation at 8,000 × *g* for 10 min at 4°C for three times after being washed and re-suspended using HEPES buffer, so the exopolysaccharides were very likely to be removed by centrifugation. *S. oneidensis* MR-1 can also produce riboflavin combining with exopolysaccharides in the process of experiment (Harald von et al., 2008), however, our previous study (Wu et al., 2014) showed that the absorbance of self-secreted riboflavin was much lower than that of the exogenous riboflavins, and the influence of self-secreted riboflavin can be ignored. In addition, Harald von et al. (2008) showed that when MR-1 was cultured with 100 mM fumarate as the electron acceptor and 50 mM lactate as the electron donor during anaerobic growth, there was nearly no riboflavin generated at the beginning, and it took 168 h for the riboflavin increasing to a highest concentration at about 0.07 μM. Because the duration time of most kinetic experiments in this study were less than 1 h, the riboflavin self-excreted by MR-1 may have very limited influences on the overall reaction kinetics. Therefore, the contribution of riboflavin combining with exopolysaccharides to Cr(VI) reduction can be ignored in this study, and the above discussion further supported that the *c*-Cyt-mediated electron transfer played a key role in Cr(VI) reduction by MR-1.

Furthermore, as the alternation of pH can also influence the Cr speciation, the speciation of Cr(VI) and Cr(III) was analyzed using Visual Minteq 3.0, which showed that results agreed with theoretical predictions (Parker et al., 2011). Under the neutral pH range tested (Borloo et al., 2007; Ross et al., 2009; Belchik et al., 2011), the dominant species of Cr(VI) are CrO_4_^2-^ and HCrO_4_; thus, the pH in the range of interest does not appear to significantly influence the species of Cr(VI). The dominant species of Cr(III) include not only the soluble species [Cr(OH)_2_^+^, Cr(OH)^2+^, and Cr(OH)_3_(aq)] but also the insoluble species [Cr(OH)_3_(s), Cr_2_O_3_], and hence, the pH in the range of interest may influence the species of Cr(III). The toxicity of Cr is due to the reactivity and solubility of the chromate anion. At circumneutral pH, Cr(VI) reduction to Cr(III) leads to its precipitation as insoluble Cr(OH)_3_. The Cr(III) showed weakened toxicity as pH dropped, probably due to its increasingly limited solubility (Rai et al., 2004; Remoundaki et al., 2007). The low Cr(VI) reduction rate at low pH may be due to the low growth and metabolism of cells, which has the most suitable pH at approximately 7 (Fein et al., 2010). The results of OD_600_ analysis further support the effects of pH on cell growth.

Regarding to the toxicity in increasing Cr(VI) concentration, while it has not been deeply investigated here, some proposed mechanisms have been reported previously. One possible mechanism is that Cr(VI) enters MR-1 cytoplasm through the sulfate transport mechanism to react with cellular DNA (Viamajala et al., 2004). A second possible mechanism is that the Cr(VI) reduction by MR-1 is self-inhibitory (Parker et al., 2011). Previous reports showed that MR-1 rapidly reduced Cr(VI) at the initial stage of reaction with 100–200 μM Cr(VI), but the cells gradually lost their ability to survive as the Cr(III) reduction product appeared (Bencheikh-Latmani et al., 2007; Gorby et al., 2008). The Cr(III) produced inside cells during Cr(VI) reduction may cross-link the phosphate backbone of DNA with peptides and amino acids such as cysteine and histidine (Zhitkovich et al., 1996), thereby inhibiting cell function and causing toxicity. The extracellular Cr(VI) reduction was not fast enough to prevent all Cr(VI) from entering the cell, Cr(III) precipitates were found outside and in the cytoplasm of the cells (Middleton et al., 2003). Also, higher concentration of Cr(VI) caused more intracellular precipitation of Cr(III) in MR-1 cell. The Cr(III) precipitates was only found outside MR-1 cells when 100 μM Cr(VI) was used as the sole terminal electron acceptor (Daulton et al., 2002). While Cr(III) precipitates were found both inside and outside MR-1 cells with 200 μM Cr(VI) (Belchik et al., 2011). A third possible mechanism of Cr(VI) toxicity to MR-1 is due to inhibition of anaerobic respiratory functions (Viamajala et al., 2004). It was manifested that anaerobic Cr(VI) reduction occurs in the electron transport pathway by cytochrome c or b along the respiratory chains in the inner membrane (Ahemad, 2014; Thatoi et al., 2014). The interaction of Cr(VI) with cytochromes might cause disruption of essential energy-deriving cell functions and result in inhibition of growth (Myers et al., 2000). Hence, with increasing concentration of Cr(VI), the mechanism for the toxicity might be attributed to Cr(VI) reacting with cellular DNA, the precipitation of Cr(III) inside the cells, and the inhibition of anaerobic respiratory functions by Cr(VI).

Based on the aforementioned results and discussion, it can be indicated that this study will have substantial implications for quantitatively evaluating the roles of *c*-Cyts in intact cells during Cr(VI) reduction processes. As *c*-Cyts play key roles in extracellular electron transfer processes, the direct measurement of *c*-Cyts reflects the real physiological and metabolic functions that take place during extracellular Cr(VI) reduction (Ross et al., 2009). In addition, despite the recent progress in describing the *c*-Cyt-mediated Cr(VI) reduction by various models, such as Michaelis–Menten model (Yamamoto et al., 1993; Shen and Wang, 1994b) and dual-enzyme model (Viamajala et al., 2003), the enzyme was not directly measured experimentally to further explore the enzymatic reactions. The *in situ* examination of redox status of *c*-Cyts will have the potential of being used to verify the outcome derived from the relevant models.

In summary, Cr(VI) and Heme_red_ were directly measured *in situ* using the DT spectra to reflect the status of Cr(VI) reduction by *c*-Cyts in intact cells, which provides a useful approach to understand the behavior of outer membrane enzymes of MR-1 under non-invasive conditions. In the presence of Cr(VI), the reduced *c*-Cyts initially rapidly decreased and then slowly recovered under all tested incubation conditions. The reduced product, Cr(III), might cause toxicity to the cells, resulting in an inhibitory effect on the Cr(VI) reduction and Heme_red_ recovery. The highest Cr(VI) reduction rate and fastest recovery of *c*-Cyts were obtained at pH 7.0 and 30°C, with sodium lactate serving as an electron donor. These conditions may be optimal due to the resemblance to suitable physiological conditions, which are favorable for metabolism and Heme_red_ recovery. The presence of O_2_ greatly inhibited Cr(VI) reduction by competing with Cr(VI) as an electron acceptor. Therefore, the method established for monitoring *in vivo* cytochrome activity and the optimization of incubation parameters will provide new insight into the microbial metal reduction processes under non-invasive conditions.

## Author Contributions

TL, RH, and FL designed the work. RH, YW, DC, and YW conducted the experiments. RH, TL, and XL wrote the manuscript.

## Conflict of Interest Statement

The authors declare that the research was conducted in the absence of any commercial or financial relationships that could be construed as a potential conflict of interest.
