# Supplementary material for: Effects of Incubation Conditions on Cr(VI) Reduction by c-type Cytochromes in Intact Shewanella oneidensis MR-1 Cells
Source: Front Microbiol. 2016 May 19;7:746. doi: 10.3389/fmicb.2016.00746 (PMC4872037; doi:10.3389/fmicb.2016.00746)
Supplement: Supplementary file 1 [file Data_Sheet_1.PDF]

Supporting information for

Effects of incubation conditions on Cr(VI) reduction by *c*-type cytochromes in intact *Shewanella oneidensis* MR-1 cells

Rui Han[1,2], Fangbai Li[2], Tongxu Liu[2], Xiaomin Li[2], Yundang Wu[2], Ying Wang[2], Dandan Chen[2]*

[1]School of Environment and Energy, South China University of Technology, Guangzhou 510006, P. R. China, [2]Guangdong Key Laboratory of Agricultural Environment Pollution Integrated Control, Guangdong Institute of Eco-Environmental and Soil Sciences, Guangzhou 510650, P. R. China

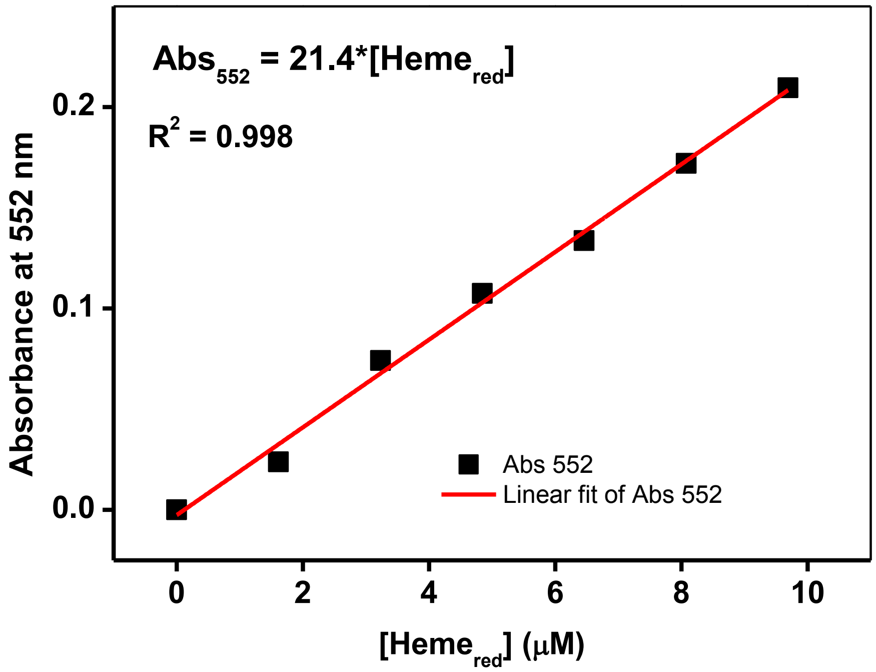

FIGURE | S1. UV/Vis diffuse-transmittance for the horse heart cytochrome *c*. Absorption values at 552 nm were plotted against [Heme_{red}].

Horse heart cytochrome c is a heme protein containing a single polypeptide chain and a single heme group with an absorbance maximum at λ_{max} = 552 nm (reduced form) and a molecular mass of 12,384 Da. The reduced (dithionite)-minus-oxidized values at 552 nm for varied concentrations of horse heart cytochrome *c* were used to plot the standard curve shown in Fig. S1. The relationship between [Heme_{ox}] in MR-1 and the absorbance at 552 nm can be expressed as shown in Eq.(S1).

$$\left[\text{Heme}_{red}\right] = \frac{\text{Abs}_{552}}{21.4} \qquad \text{(S1)}$$

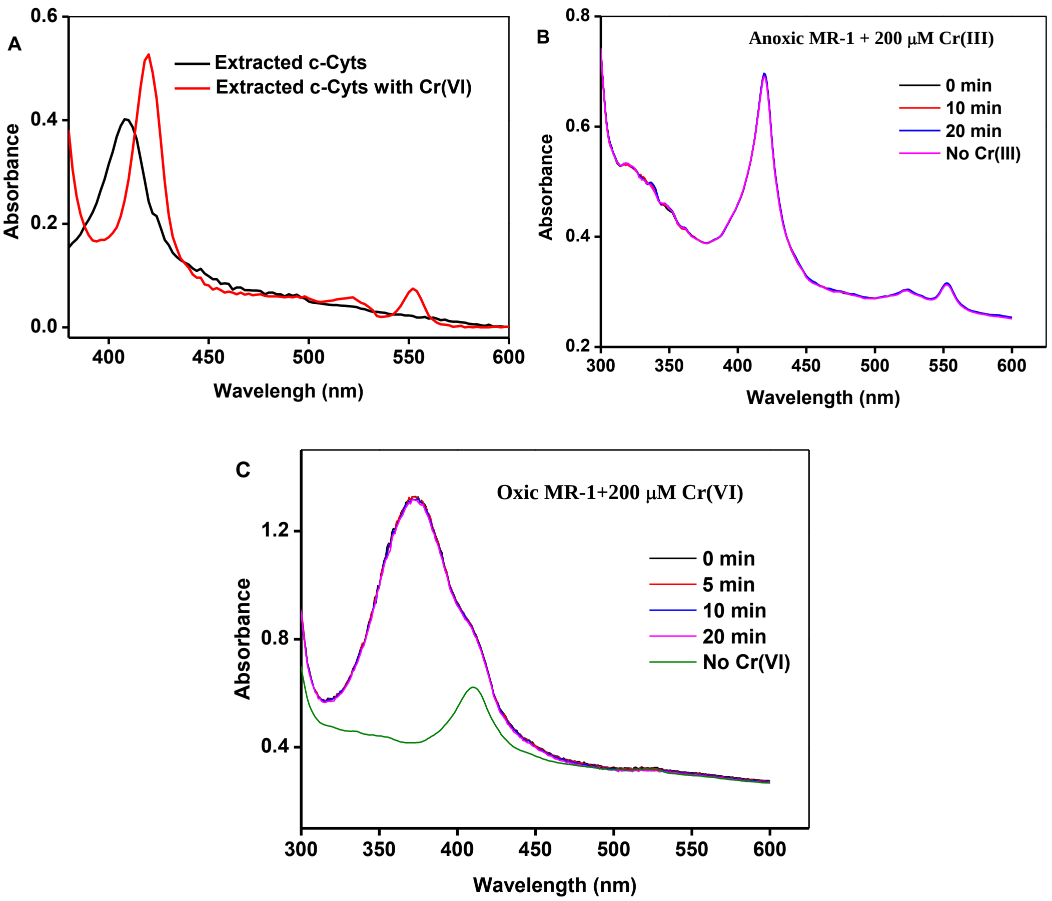

FIGURE S2 | The kinetic spectra of (A) extracted proteins of MR-1 before and after addition of Cr(VI) (50 μM), (B) the intact MR-1 cell suspensions with Cr(III) (200 μM) incubated under anoxic conditions for different times, and (C) the intact MR-1 cell suspensions with Cr(VI) (200 μM) incubated under oxic conditions for different times. Cell density of MR-1: 1.07×10^{12} cells mL^{-1}.

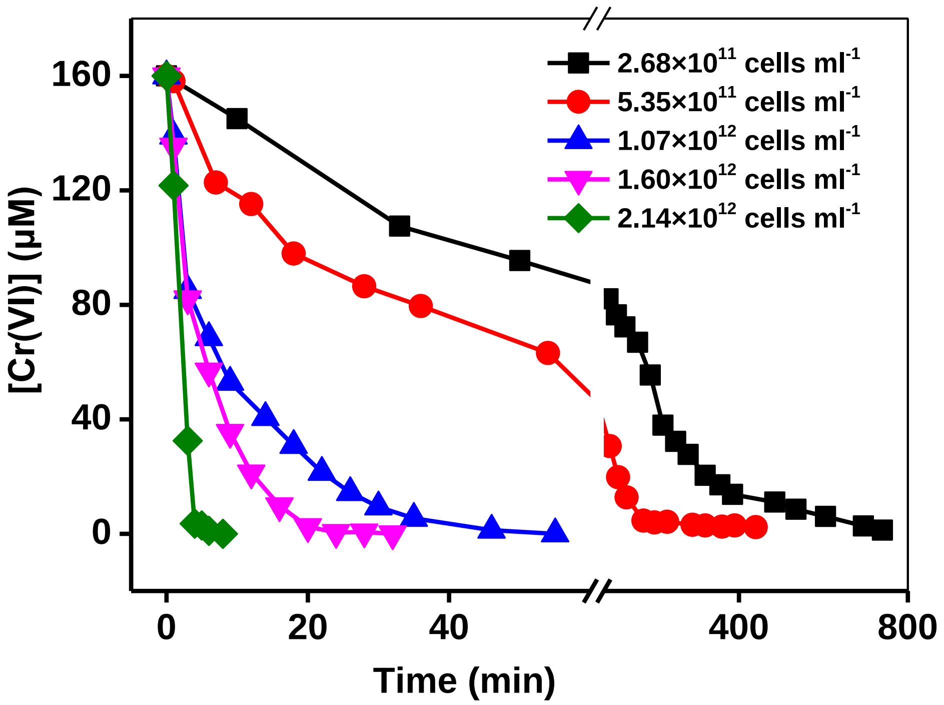

FIGURE S3 | The kinetics of Cr(VI) reduction by *c*-Cyts in intact MR-1 cell suspension for MR-1 cells with densities ranging from 2.68×10^{11} cells mL^{-1} to 2.14×10^{12} cells mL^{-1}. All experiments were conducted with 160 μM Cr(VI) and 20 mM sodium lactate.

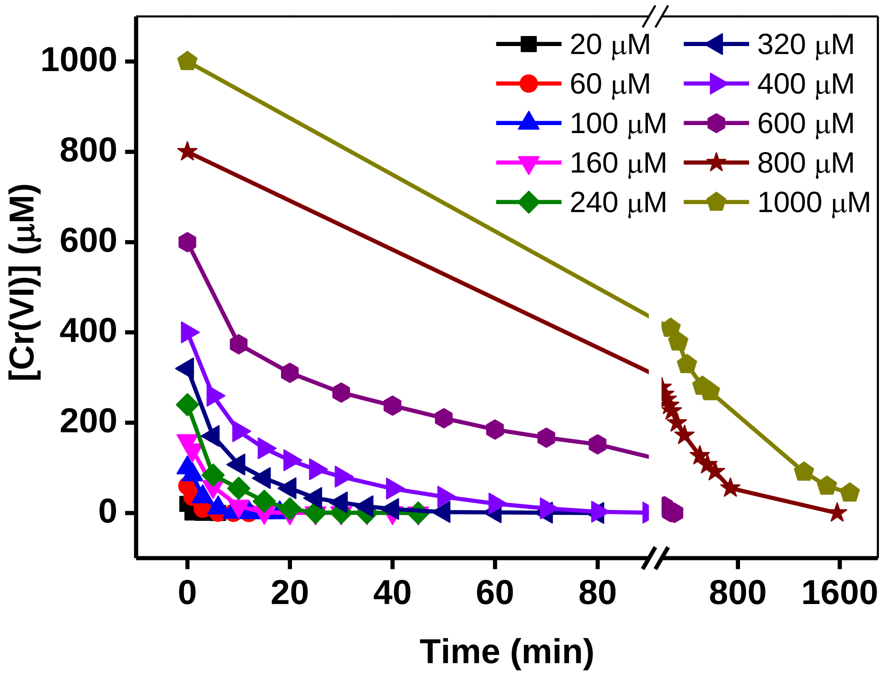

FIGURE S4 | The kinetics of Cr(VI) reduction by c-Cyt in intact MR-1 cell suspension for Cr(VI) concentrations ranging from 20 μM to 1000 μM. MR-1: 1.07×10^{12} cells mL^{-1}.

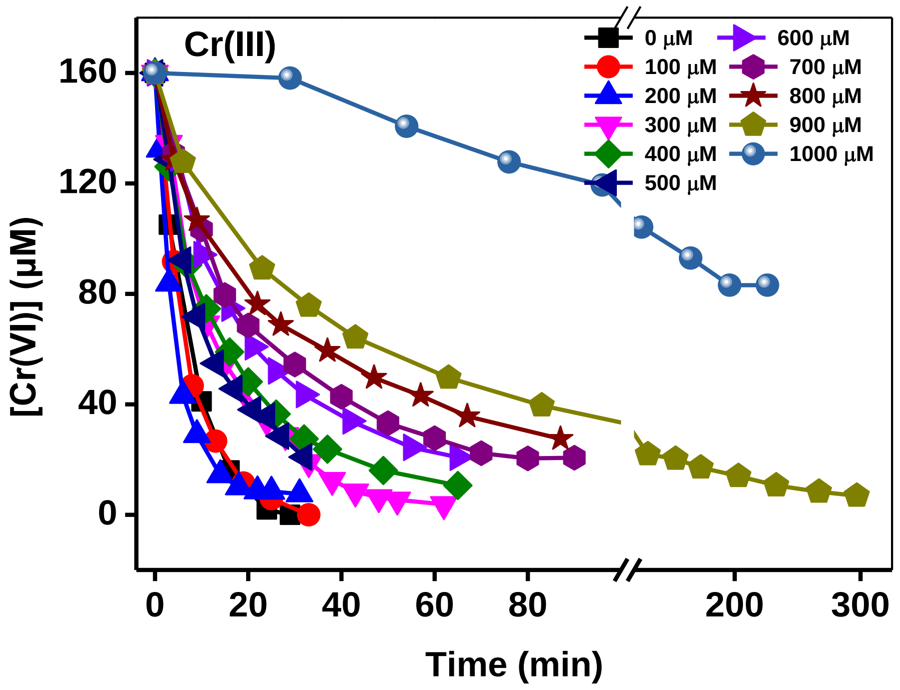

FIGURE S5 | The kinetics of Cr(VI) reduction by *c*-Cyts in intact MR-1 cell suspension for Cr(III) concentrations ranging from 100 μM to 1000 μM. All experiments were conducted with 160 μM Cr(VI), MR-1: 1.07×10^{12} cells mL^{-1}, and 20 mM sodium lactate.

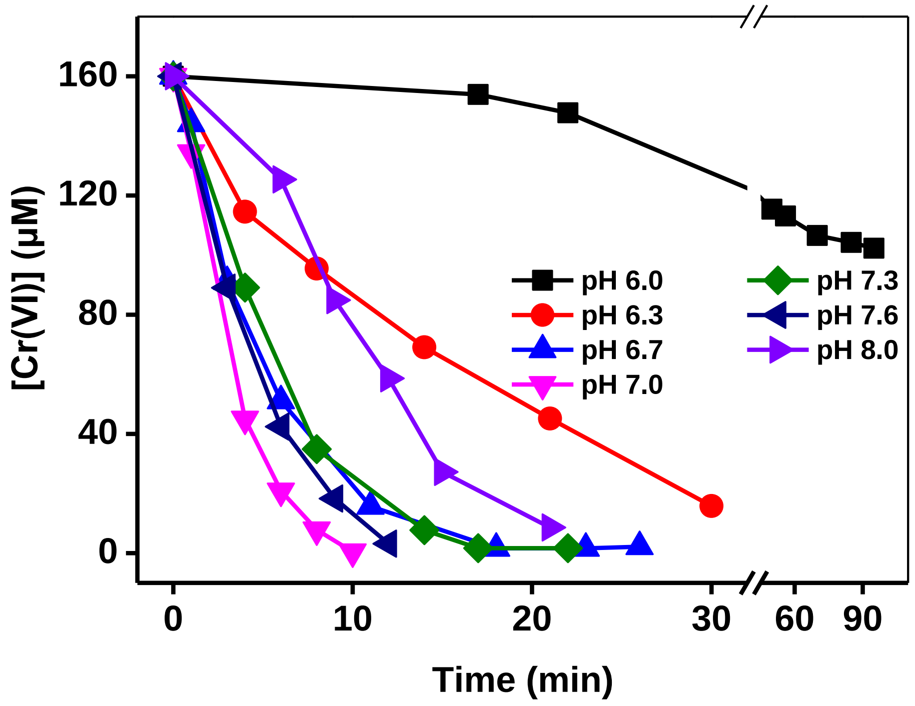

FIGURE S6 | The kinetics of Cr(VI) reduction by *c*-Cyts in intact MR-1 cell suspension at pH conditions ranging from pH 6.0 to pH 8.0. All experiments were conducted with 160 μM Cr(VI), MR-1: 1.07×10^{12} cells mL^{-1}, and 20 mM sodium lactate.

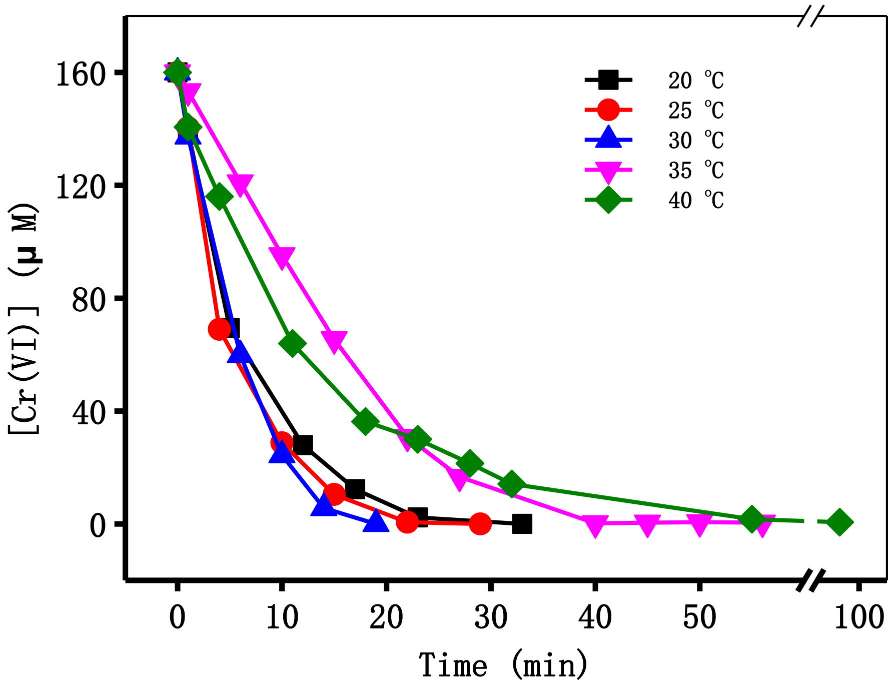

FIGURE S7 | The kinetics of Cr(VI) reduction by *c*-Cyts in intact MR-1 cell suspension at various temperatures. All experiments were conducted with 160 μM Cr(VI), MR-1: 1.07×10^{12} cells mL^{-1}, and 20 mM sodium lactate.

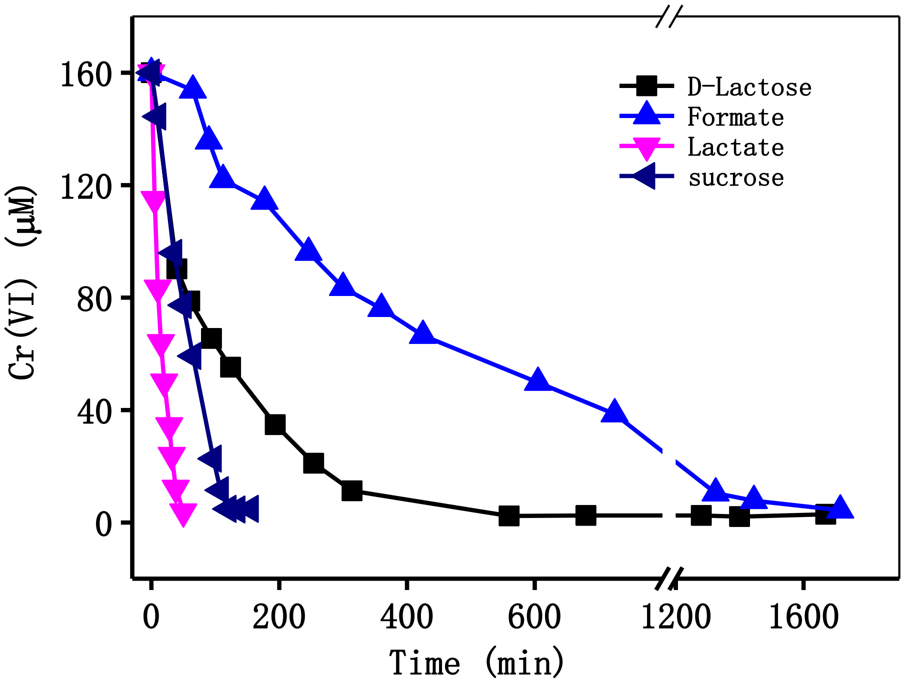

FIGURE S8 | The kinetics of Cr(VI) reduction by *c*-Cyts in intact MR-1 cell suspension in the presence of different electron donors. All experiments were conducted with 160 μM Cr(VI), MR-1: 1.07×10^{12} cells mL^{-1}, and 20 mM of each electron donor.

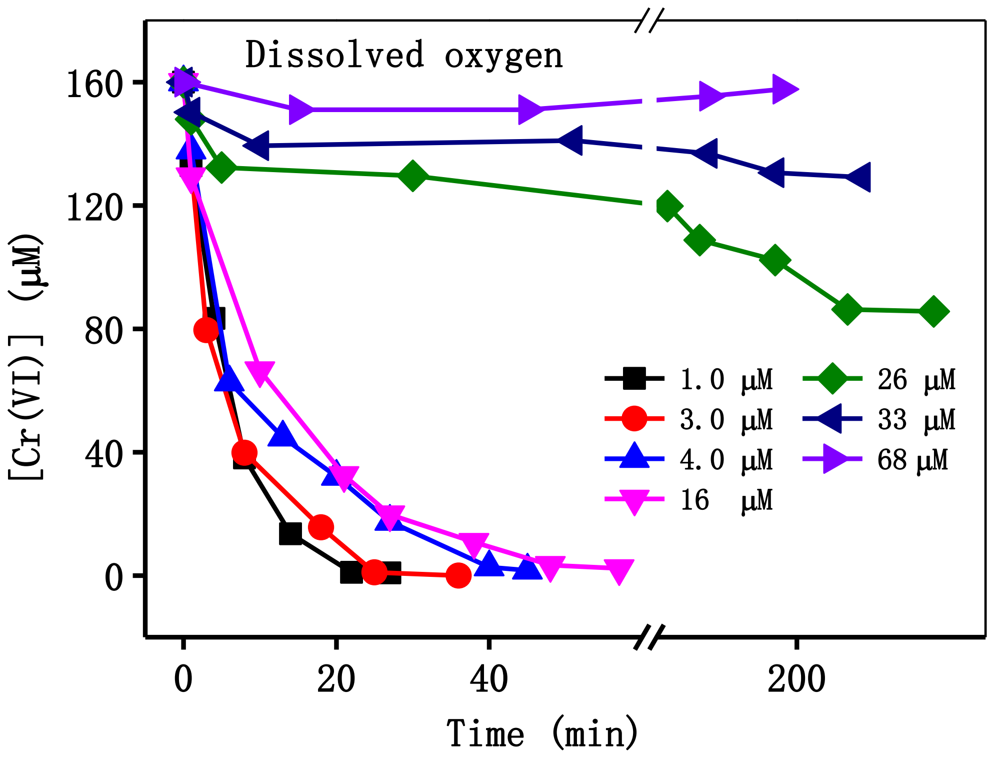

FIGURE S9 | The kinetics of Cr(VI) reduction by *c*-Cyts in intact MR-1 cell suspension with dissolved oxygen concentrations ranging from 1.0 μM to 68 μM. All experiments were conducted with 160 μM Cr(VI), MR-1: 1.07×10^{12} cells mL^{-1}, and 20 mM sodium lactate.
